# Helmet Use and Associated Factors among Thai Motorcyclists during Songkran Festival 

**DOI:** 10.3390/ijerph9093286

**Published:** 2012-09-10

**Authors:** Penprapa Siviroj, Karl Peltzer, Supa Pengpid, Sompong Morarit

**Affiliations:** 1 Department of Community Medicine, Chiang Mai University, Chiang Mai 50200, Thailand; Email: psiviroj@mail.med.cmu.ac.th; 2 HIV/AIDS/SIT/and TB (HAST), Human Sciences Research Council, Pretoria 0001, South Africa; 3 Department of Psychology, University of the Free State, Bloemfontein 9300, South Africa; 4 Department of Health System Management and Policy, University of Limpopo, Medunsa Campus, Pretoria 0204, South Africa; Email: supaprom@yahoo.com; 5 Health Promotion Region 10, Ministry of Public Health, Chiang Mai 5000, Thailand; Email: smorarit@gmail.com

**Keywords:** helmet use, motorcyclists, road safety awareness campaign, Thailand

## Abstract

The aim of this study was to assess helmet use and associated factors among motorcycle riders during Songkran festival in Thailand. A cross-sectional survey was conducted to determine the prevalence of helmet use among Thai motorcycle riders (sample size = 18,998) during four days of the Songkran festival. For this sample, the population of motorcycle riders was consecutively selected using quota sampling from 12 petrol stations in four provinces from each of the four main geographical regions of Thailand. The study was conducted at petrol stations at roads in town, outside town and highway at different time intervals when trained field staff administered a structured questionnaire and performed an observation checklist. Results indicate that 44.2% of the motorcycle riders and 72.5% of the motorcycle passengers had not been using a helmet. In multivariable analysis demographics, environmental factors, helmet use experiences and attitudes and recalling a lower exposure to road safety awareness (RSA) campaign were associated with non-helmet use among motorcyclists. It appears that the RSA campaign may have some positive effect on reducing non-helmet use among motorcycle riders during the Songkran festival.

## 1. Introduction

The Road Traffic Injury (RTI) fatality rate in Thailand was 40 per 100,000 population in 2007, *i.e*., double the world average for low- and middle-income countries [1]. In Thailand, RTIs are the second leading cause of burden of disease [2]. Motorcycle-related crashes accounted for the majority (more than 70%) of injuries and deaths related to road traffic in Thailand [3,4,5]. A number of known behavioural risk factors have been identified, *i.e.*, drunk driving, speeding, substance abuse, and failure to use helmets and seat belts [6,7]. Youth risk survey data from Bangkok show that the risk behaviours leading to traffic accidents were rarely or never having worn a helmet while motorcycling (50.1%) [8]. Survey data from 38 provinces in Thailand in the year 2007 found that the helmet wearing rate in drivers was 54.0 % and among passengers 30.9% [9]. Other studies from low and middle income countries also found a high prevalence of non-helmet use of motorcyclists (China 34–37.4% [10,11], India 68.6% [12], Iran 89.3% [13], Nigeria 76.2% [14], Vietnam 70.1%, [15] and generally lower among passengers (China 65.9% [11]). Based on a literature review, it was found that factors associated with non-helmet use among motorcyclists included age (younger people) [10,15,16], gender (male riders [10], female riders [11,15]), being a passenger [11], location (secondary streets [10], inner-city roads [15], outside city roads [11]), time of day (less during evening, night-time [10,16,17,18], weekends [10], lack of intention and perceived behavioural control [13] and having ever had a traffic accident [19].

According to the Road Traffic Act 1979, section 122, motorcyclist and passenger are obliged to wear a helmet to protect themselves from harm during driving [9]. Helmets and helmet use laws have been shown to be effective in reducing head injuries and deaths from motorcycle crashes [20]. In a study in Thailand, the probability of fatality due to head injuries was reduced by 38% for motorcyclists wearing helmets and by 43% for those riding under alcohol influence [18]. Ichikawa *et al.* [17] found that after enforcement of the helmet act, helmet-wearers increased five-fold while head injuries decreased by 41.4% and deaths by 20.8% in Thailand. Motorcycle rider education seemed to have had a positive effect on changes in risk behaviours and motorcycle-related injuries in rural Thailand [21].

From 1997 an active public education programme was undertaken on a national scale to raise awareness about road safety and to support law enforcement. This included dissemination of knowledge through multiple channels, e.g., roadside posters, stickers on the back of vehicles, sporadic radio and TV programmes or spots, public announcements and press reports [22]. After 2000, communication about the law was increased and both governmental and nongovernmental agencies started to participate in traffic injury prevention and control programmes including helmet wearing among motorcyclists and passengers [6,23]. This included also increased Road Safety Awareness (RSA) campaigns during the Songkran festival, but seemingly not everywhere the full range of RSA campaigns were implemented [24]. In addition, the following law enforcement strengthening activities focused on helmet-use, safe motorcycle-riding, driving licenses, and speed limiting especially during the Songkran Festival, and a “drive safely, turn head-light and wear helmet” campaign [25]. 

Songkran is the New Year celebration in Thailand, set by the solar calendar since ancient times. It takes place between 13 and 15 April. Songkran festivals are major holidays that encourage a million travellers who travel to/from their hometown and doing the activities during these holiday periods [26]. Unfortunately, the number of road accidents, fatalities and injuries increases dramatically; in April the number of road traffic fatalities was almost 1,200 persons, way above the average of less than 1,000 [26]. The daily fatalities during Songkran festival rise up to 84 and 95 persons per day, compared with an average of 34 persons per day in the non-festival period. Similarly, daily injuries during Songkran holidays in April increased to 4,900 and 5,650 persons, or 98% and 128% compared with an average of 2,468 persons per day during the non-festival period [27]. During Songkran holidays motorcycles were the major cause of road traffic accidents (76.7%) [28] and the most common causes identified included alcohol use (41%) [28,29] and not wearing a helmet (98.4%) [28]. The aim of this study was to assess helmet use and associated factors among motorcycle riders during Songkran festival in Thailand.

## 2. Methodology

### 2.1. Sample and Procedure

A cross-sectional survey was conducted to determine the prevalence of helmet use among Thai motorcycle riders. The recruitment period of this project was during four days of the Songkran festival for 13–16 April, 2007. For this sample the population of motorcycle riders from 12 petrol stations was selected using quota sampling from four provinces from each of the four main geographical regions of Thailand excluding Bangkok. Provinces were Chiang Mai, Lampang, Nakhon Sawan and Phichit in the northern region, Nakhon Ratchasima, Khon Kaen, Udon Thani, and Loei in the northeastern region, Songkhla, Phuket, Surat Thani, and Trang in the southern region, and Phra Nakhon Si Ayutthaya, Chonburi, Chachoengsao, and Phetchaburi in the central region. In total 48 petrol stations (three petrol stations per province) were selected. In town, the petrol station on the road with the largest shopping mall was selected; out of town the petrol station on the road leading to the largest district was selected; in terms of petrol station on the highway, each province only has one highway. If there was more than one petrol station on the selected road or highway, the largest petrol station was selected. The study team spent four days at each petrol station road venue (roads in town, outside town and highway) for 7:00–9:00, 13:00–15:00, 17:00–19:00, and 22:00–24:00. 

All consecutive motorcycle riders who entered the petrol station were asked to participate by trained personnel (who were students from Chiang Mai University that were trained by the research team) while they were having their gas tank filled. The number of vehicles and time interval for vehicle selection were determined by the availability of field staff to conduct a motorcycle rider observation, interview and alcohol test. The target sample size was 100 motorcycle riders from each of the petrol stations per time period, except during 22:00–24:00 for which 50 motorcycle riders were targeted. Trained field staff administered a structured questionnaire and performed an observation checklist. The project was approved by the Ethics Committee for research in human subjects of the public health programme, Chiang Mai University.

### 2.2. Measures

The primary outcome of the study was helmet use. Helmet use was assessed by observation. The questionnaire covered demographic data, motorcycle characteristics, history of road traffic accidents, known risk factors such as age, sex, environmental factors, helmet use experiences and attitudes, and exposure to road safety awareness (RSA) campaign.

### 2.3. Data Analysis

Data were analyzed using Statistical Package for the Social Sciences (SPSS) for Windows software application programme version 19.0. Frequencies, means, and standard deviations were calculated to describe the sample. Data were checked for normality distribution and outliers. Interaction between predictor variables was also examined and it was found that none of the variables had a Variance Inflation Factor (VIF) value above 2.5. Associations of non-helmet use were identified using logistic regression analyses. A multivariable regression model was constructed. Independent variables (demographics, environmental factors, helmet use experiences and attitudes and exposure to road safety awareness campaign) from the univariate analyses were entered into the multivariable model if significant at *P* < 0.05 level. For the model, the R^2^ is presented to describe the amount of variance explained by the multivariable model. Probability below 0.05 was regarded as statistically significant.

## 3. Results and Discussion

### 3.1. Sample Characteristics

The sample included 18,998 motorcycle riders (67.3% male and 32.7% female) of whom 320 refused to participate, giving a response rate of 98.3%. Overall, 44.2% of the motorcycle riders had not been using a helmet. Almost half of the motorcycle riders (49.6%) had a passenger (60.2% female and 39.8% male) of which 72.5% had not been wearing a helmet. The largest group of the motorcycle riders were between 18 to 25 years old (43.9%), followed by 26 to 59 years olds (41.8%), 2.2% were below the legal motorcycle riding age (<15 years), and among those who were 15 to 17 years old 46.1% were illegally riding a size of motorcycle with more 110 cc. The data collection was equally distributed across four regions, four days of data collection; three locations of data collection and data collection across three different times during the day, only data collection in the evening yielded a smaller sample (see Table 1). 

### 3.2. Helmet Use Experiences, Attitudes and Road Safety Awareness Campaign Exposure

About one third of the motorcycle riders (33.4%) indicated that they had been in an accident before, mostly as a rider (75.5%), followed by passenger (22.8%) and pedestrian (1.5%). Almost half had had the intention to use a helmet (44.8%). A large group agreed with the danger of non-helmet use (75.0%) and almost two-thirds were highly aware of the danger of not using a helmet (55.8%). Almost half (47.2%) had been caught with the non-use of a helmet before. 

The majority (83.9%) had heard about an RSA campaign, 29.2% had frequently heard or seen the RSA campaign on the radio and/or TV, and 29.3% frequently followed TV news reports on road traffic injury (RTI) statistics. Two in five (40.1%) had also been talking to others about the RSA campaign and most (89.0%) liked the RSA campaign (see Table 2).

**Table 1:** Demographic, motorcycle and environmental sample characteristics of motorcyclists (during Songkran festival) (N = 18,998).

	Total		Non-helmet use of rider	
	Number	%	Number	%
**Demographics**				
All	18,998		8,369	44.2
Male	12,744	67.3	5,683	44.7
Female	6,183	32.7	2,651	43.0
*Age (by self-report)*				
<15	403	2.2	203	50.6
15–17	2,045	11.4	1,245	61.1
18–25	7,860	43.9	3,895	49.7
26–59	7,480	41.8	2,449	32.8
60 or more	128	0.7	39	31.0
*Region *				
North	4,546	23.9	2,850	62.9
Central	4,663	24.5	2,003	43.1
Northeast	4,989	26.3	2,135	43.1
South	4,800	25.3	1,381	28.8
**Motorcycle and environmental characteristics**				
*Type of motorcycle*				
50–110 cc	7,047	31.1	3,179	45.2
Over 110 cc	5,910	37.1	2,717	46.1
Missing	6,041	31.8		
*Data collection time*				
07:00–09:00	5,557	29.3	2,308	41.7
13:00–15:00	5,790	30.5	2,619	45.4
17:00–19:00	5,688	30.0	2,524	44.5
22:00–24:00	1,943	10.2	910	47.1
*Date of data collection*				
13 April 2007	4,910	25.8	2,313	47.3
14 April	4,616	24.3	2,068	44.9
15 April	4,744	25.0	2,217	47.0
16 April 2007	4,726	24.9	1,770	37.6
*Location of data collection*				
Main road in town	6,306	33.2	2,663	42.4
Roads out of town	6,265	33.0	2,872	46.1
Highway	6,406	33.8	2,815	44.2

**Table 2:** Helmet use experiences and attitudes and exposure to road safety awareness campaign of motorcyclists (during Songkran festival) (N = 18,998).

Variable	Response options	Total motorcycle riders		Non-helmet user	
		Number	%	Number	%
**Helmet use experiences and attitudes**					
Been in accident before	No	12,649	66.6	5,479	43.5
	Yes	6,349	33.4	2,890	45.7
Rider status when in accident	Rider	4,557	75.7	2,146	47.2
	Passenger	1,370	22.8	601	44.1
	Pedestrian	93	1.5	37	39.8
Have you ever used a helmet before	No	7,600	40.2	5,925	52.6
	Yes	11,290	59.8	2,395	31.7
Perceived danger of not using a helmet	No	4,720	25.0	2,008	42.7
	Yes	14,175	75.0	6,316	44.7
Aware of danger of not using a helmet	Low	840	4.4	425	50.7
	Moderate	7,525	39.8	3,328	44.4
	High	10,544	55.8	4,580	43.6
Perceived risk about being caught by the police because of not using a helmet	High risk	7,788	41.3	3,602	46.4
	Moderate risk	7,411	39.3	3,271	44.3
	Low risk	2,078	11.0	855	41.3
	No risk	1,595	8.5	584	36.7
Ever been caught by police because of not using a helmet	No	8,918	52.8	3,839	38.6
	Yes	9,983	47.2	4,483	50.4
**Exposure to road safety awareness (RSA) campaign**					
Heard advertising campaign on RSA	No	3,066	16.1	1,428	46.9
	Yes	15,926	83.9	6,938	43.7
RTI campaign on radio or TV	Never	2,476	13.2	1,198	48.5
	Not often	10,253	54.5	4,391	43.0
	Frequently	5,493	29.2	2,409	44.0
	Not sure	587	3.1	272	46.4
Talking to others about RSA in the media	Never	7,729	40.8	3,789	49.2
	Ever	7,600	40.1	3,031	40.0
	Not sure	3,620	19.1	1,533	42.6
How feel about RSA media	Not like	1,119	5.9	560	50.0
	Like a little bit	11,676	61.7	5,148	44.3
	Like very	5,173	27.3	2,205	42.7
	much	955	5.0	433	45.4
	Not sure				
Follows the TV news report on RSA statistics	Never	2,614	13.8	1,256	48.2
	Not often	9,841	52.0	4,415	45.0
	Frequently	5,552	29.3	2,244	40.6
	Not sure	912	4.8	436	48.2

### 3.3. Association between Non-Helmet Use and Demographics, Experiences, Attitudes and RSA Campaign Exposure

In multivariable analysis, it was found that the highest proportion of non-helmet use among motorcyclists was in the age group 15 to 17 years old and in riders from the northern region in Thailand. Motorcyclists who were having a passenger were significantly more often not using helmet than those who had no passenger (see Table 3).

**Table 3:** Results: Association between non-helmet use and demographics, environmental factors, helmet use experiences and attitudes and RSA campaign exposure (during Songkran festival) by the logistic regressions.

		Non-helmet use-rider
		Adjusted Odds Ratio ^a^
**Demographics**	*Gender*	
	Female	1.00 (Reference)
	Male	1.00 (0.93–1.07)
	*Age*	
	<15 years old	1.00
	15–17	1.45 (1.16–1.83)***
	18–25	0.95 (0.77–1.18)
	26–59	0.46 (0.37–0.57)***
	60 or more	0.33 (0.21–0.52)***
	*Region*	
	North	1.00
	Central	0.40 (0.36–0.44)***
	Northeast	0.34 (0.31–0.37)***
	South	0.19 (0.18–0.21)***
**Environmental factors**	Motorcycle up to 110 cc *vs.* more than 110 cc	0.92 (0.87–1.14)
	Having a passenger *versus* none	1.62 (1.52–1.73)***
	*Day of Songkran festival*	
	13 April 2007	1.00
	14 April	0.93 (0.85–1.01)
	15 April	1.03 (0.94–1.12)
	16 April 2007	0.70 (0.64–0.77)***
	*Time of the day*	
	07:00–09:00	1.00
	13:00–15:00	1.15 (1.06–1.25)***
	17:00–19:00	1.16 (1.07–1.26)***
	22:00–24:00	1.27 (1.13–1.43)***
	*Type of road*	
	Main road in town	1.00
	Roads out of town	1.20 (1.11–1.30)***
	Highway	1.10 (1.03–1.21)*
**Helmet use experiences and attitudes**	Been in accident before	1.08 (1.01–1.16)*
	*Awareness of danger of non-helmet use*	
	High	1.00
	Moderate	1.09 (0.93–1.27)
	No/not sure	1.10 (1.03–1.18)**
	Caught with non-helmet use	1.55 (1.45–1.66)***
**Exposure to road safety awareness campaign**	Not heard about RSA campaign	1.11 (1.00–1.23)*
	*Frequency of exposure to RSA campaign*	
	Frequently	1.00
	Not often	0.98 (0.91–1.06)
	Never/not sure	1.13 (1.00–1.27)*
	Not talking to others about RSA media	1.02 (0.96–1.10)
	*Follows RTI statistics*	
	Frequently	1.00
	Not often	1.07 (0.99–1.16)
	Never, not sure	1.13 (1.02–1.25)*

^a ^Hosmer & Lemeshow Chi-square = 21.70, *P* = 0.005; Log likelihood: 21,273.24, Nagelkerke R^2^: 0.19; ***P < 0.001; **P < 0.01; *P < 0.05; number of observations of this regression = 18,850.

Non-helmet use was found to be more frequent earlier during the Songkran festival, later during the day, on roads out of town and on highways. Respondents who had been in an accident before, had low awareness of the danger of non-helmet use and having been caught for non-helmet use were more likely to not wear a helmet. Motorcyclists who recalled a lower exposure to road safety awareness campaign (not heard of RSA, never exposed to RSA and did not follow RTI statistics) were more likely not to wear a helmet compared to those who recalled a higher exposure to road safety awareness campaign. 

### 3.4. Discussion

This study among a large sample of motorcyclists in Thailand found that 44.2% of the motorcycle riders and 72.5% of motorcycle passengers had not been wearing a helmet, which is similar to a survey of 2007 in Thailand, with 46% and 69.1% non-helmet use among motorcyclists and passengers, respectively [9]. Similar high rates of non-helmet use among motorcycle riders and passengers have been found in some other studies in low and middle income countries [8,10,11], and even lower rates of helmet use was found in countries such as India [12], Iran [13], Nigeria [14] and Vietnam [15]. These findings highlight that safety helmets continue to be underused by a large segment of motorcyclists in low and middle income countries. Efforts to identify solutions to increase helmet use in these parts of the world need to continue. The study found in agreement with other studies [19] that having been in an accident before, low awareness of danger of non-helmet use [13] and having a passenger [30] were associated with unhelmeted motorcycle riding. Moreover, having been caught for non-helmet use was also found to be associated with non-helmet use in this study. These findings suggest a pattern of persistent high risk behaviour among persons previously in an accident and having been caught for non-helmet use [31]. The relatively high prevalence of riding unhelmeted later in the day or at night among motorcyclists in this study is perhaps attributable to insufficient law enforcement at night. In a questionnaire survey in Indonesia, respondents reported that they were less likely to wear helmets at night when there were no police officers on the road [32]. Further, the study found in line with some other studies that demographic characteristics (younger people [10,15,16] and being a passenger [11]) were associated with non-helmet use. Environmental characteristics (earlier during the Songkran festival, later during the day or at night, coming from the Northern region, on roads out of town and on highways) [10,11,22] were also associated with unhelmeted motorcycle riding. Younger people in their teens tend to partake in high-risk behaviours such as non-helmet use [33]. Reasons for the environmental differences found may be that the enforcement by police could be greater on national highways and principal arteries and during work days and rush hour [10]. Differences may also be due to lower vehicle speeds in lateral streets or shorter driving distances, which have previously been found to be associated with low helmet use [34]. In addition, earlier during the Songkran festival and coming from the Northern region were found to be associated with lower helmet use. It is possible that helmet use increased towards the end of the Songkran festival since motorcyclists faced stricter helmet use law reinforcement. The finding of low helmet use in the Northern region in Thailand was confirmed by a study by Pitaktong *et al*. [19] who found 72.7% of male and 64.4% of female students reported non-helmet use. The results derived from studying these factors provide documentation for the priorities for campaigns or measures targeting extensive helmet use by motorcycle riders.

In contrast to other studies, this study did not find any association between gender [10,11,15] and lack of intention to use a helmet [13] and non-helmet use. Importantly, recalling a lower exposure to RSA campaign was found in this study to be weakly associated to non-helmet use among motorcyclists. This finding may indicate that the RSA campaign may have some positive effect on reducing non-helmet use among motorcycle riders during the Songkran festival. Phillips *et al.* [35] found from meta-analysis (67 studies) the weighted average effect of road safety campaigns was a 9% reduction in accidents.

### 3.5. Study Limitations

Caution should be taken when interpreting the results of this study because of certain limitations. Since the sampling procedure was not truly random, this may be a limitation of the study. As this is a cross-sectional study, causality between the compared variables cannot be concluded. A further limitation is that some variables were assessed by self-report, and desirable responses may have been given. Other examples of limitations include that substance use (alcohol and illicit drugs) [36,37,38], unfavorable weather conditions [39] and after midnight ride, experience/duration after having riding license [40] and safety riding training [41] were not assessed. Finally, the assessment of the exposure to the RSA campaign and effects on helmet use were not assessed in a controlled design, which limits the findings effects. Further, evaluating interventions by assessing the correlation between behaviours and the recall of having seen an advertisement is a finding with a high risk of confounding, as those who comply with the behaviour encouraged/enforced might be more likely to remember it (or accept being reminded about it).

## 4. Conclusions

Rates of non-helmet use by Thai motorcycle riders and passengers during Songkran festival seemed to be high. It appears that the road safety awareness campaign may have a slight positive effect on reducing non-helmet use among motorcycle riders during the Songkran festival. The presented information concerning different peaks of unhelmeted motorcyclists will be helpful in devising specific countermeasures against such risky behaviour.
